# Determinants of Revolving Door in an Acute Psychiatric Ward for Prison Inmates

**DOI:** 10.3389/fpsyt.2021.626773

**Published:** 2021-04-15

**Authors:** Isabella D'Orta, François R. Herrmann, Panteleimon Giannakopoulos

**Affiliations:** ^1^Division of Institutional Measures, Medical Direction, Geneva University Hospitals, Geneva, Switzerland; ^2^Department of Rehabilitation and Geriatrics, Geneva University Hospitals and University of Geneva, Geneva, Switzerland; ^3^Department of Psychiatry, University of Geneva, Geneva, Switzerland

**Keywords:** criminal status, prison, psychiatry, personality, revolving door

## Abstract

Among the different types of heavy use of mental health services, frequent inpatient admission in acute care units of individuals unable to return to their usual environment refers to as revolving-door (RD). RD in prisoners is related to increased violence (acted and supported) and suicidal recidivism. We explored the determinants of RD in 200 inmates from the Swiss-French speaking areas who were admitted to the sole acute psychiatric care unit for all of the Swiss-French counties, located in Geneva. The Cuzick's test for trend across ordered groups, Kruskal-Wallis test and oneway ANOVA were used to compare demographic and clinical variables between single (one admission, *N* = 100), frequent (3–7, *N* = 69) and RD (more than 8, *N* = 31) during a 12 months period. In addition, univariate and multivariable ordered logistic regression modes were built to examine the determinants of RD. The sample included 27 women (mean age: 31.2 years) and 173 men (34.5 years) who were admitted during the period 2014–2019. The vast majority were single (65%) with low level of education (<6 years, 78%). Suicidal behavior was the more frequent reason for admission (57%). Psychiatric history was positive in 77.5% of cases and in 54.5% of cases there was at least one episode of inpatient psychiatric care. The more frequent ICD-10 psychiatric diagnosis in the last admission were psychotic disorder (38%), personality disorder (29.5%) and adjustment disorder (19.5%). In contrast, depressive episodes (7%) and bipolar disorder (4.5%) were rare. Group comparison showed that the presence of court-ordered treatments, suicidal behavior, personality and psychotic disorders was associated with significantly increased frequency of RD use. In univariate models, the same factors were positively associated with RD, the highest odds ratio being found for court-ordered treatments (5.77) and personality disorders (2.14). In contrast, the diagnosis of adjustment disorders was related to decreased RD use (OR 0.25). Court-ordered treatments and personality disorders were the only factors to predict RD in multivariable regression models. These findings suggest that acute psychiatric care in these patients did not depend of environmental stressors but rather represents the expression of a long-lasting vulnerability related to their psychological profile and criminal status.

## Introduction

Heavy use of mental health services refers to the disproportionate consumption of psychiatric care and is often associated with significant cost increase and team frustration (1). There is no consensus regarding the exact definition of heavy use that may take into account the number of hospitalisations, their length as well as the number of days without inpatient or outpatient care in a given time period (2, 3). Among the different types of heavy use, frequent admission in psychiatric unit refers to as revolving-door (RD), a notion that was first described in early 70's following the closure of the psychiatric asylums. RD indicates repeated hospitalizations of patients unable to sustain an independent life in the community (4, 5). This phenomenon accounts for almost 16% of hospital days but concerns < 3% of psychiatric inpatients (6). RD did not depend on the presence of outpatient settings able to assume community-oriented care (5). Even in countries with a long-standing community-based mental health system, a small but substantial part of patients fall into this category (7). Repeated admissions to a psychiatric facility are considered as a poor outcome, since they have a negative impact on patient well-being and mortality and dramatically rises mental health-related costs (8, 9). Despite the fact that RD is both an economical and quality issue in psychiatry (8), its clinical and social determinants remain poorly explored. It has been long considered that crisis discharge due to lack of beds was the main determinant of this condition (10, 11). However, RD also occurs in the absence of significant bed pressure implying the presence of mental health policy-independent determinants (12). Psychotic disorders (mainly schizophrenia diagnosis), substance use (associated with psychotic and depressive disorders), obsessive-compulsive disorder and alcohol dependence, as well as borderline personality disorder were all associated with RD. In particular, drug addiction associated with schizophrenia or anxious disorders was related to increased use of acute psychiatric wards (13). In bipolar patients, RD was associated with mixed episodes and medical comorbidities (14). Lack of therapeutic alliance and treatment discontinuation are thought to increase RD, mainly in cases with concomitant substance abuse (15, 16). However, the relative contribution of these parameters varies substantially as a function of care setting and population of reference (17–21). Although poor socio-economic status and compulsory admissions are not related to RD in most previous studies (1, 17, 22–24), being unemployed and/or living in a residential facility (24) or having severe social disability (25), may lead to RD use of psychiatric facilities. Environmental factors can also influence this phenomenon: an urban environment (12) and, even more, family conflicts (26) could increase the tendency for repeated hospitalizations.

The RD also concerns prisoners with acute psychiatric disorders, a growing population in western countries. High levels of psychiatric morbidity are well-documented in prisoners and are frequently associated with violence, victimization and self-harm (27). There is evidence that, in some countries the prevalence of mental health disorders among prisoners is even higher than in psychiatric facilities, yet they remain poorly diagnosed and treated (28). Common psychiatric disorders such as anxiety disorder and depression are over-represented, as well as psychotic disorders and drug misuse. RD in prisoners is a very sensitive issue since it is closely related to increased violence, both acted and supported, as well as suicidal recidivism. To our knowledge, no studies explored the demographic, social and clinical determinants of RD in this population. We had the opportunity to address this issue in a large sample of prison inmates from the Swiss French speaking areas who were admitted to the sole acute psychiatric care unit for all of the Swiss French counties, located in Geneva.

## Materials and Methods

### Study Sample and Data Collection

We examined the psychiatric records corresponding to all admissions during a 5-year period (2014–2019) in UHPP (Unité hospitalière de psychiatrie penitentiaire), a forensic psychiatry unit of 15 beds specially designed for acute psychiatric care of inmates. It is located in a forensic penitentiary (referred to as Curabilis), a complex facility that also includes five units for prisoners with court-ordered inpatient treatments for severe mental disorders. The catchment area includes all of the prisons of French and Italian speaking counties in Switzerland. Admissions may be voluntary or compulsory. The total mean number of admissions per year for the period of reference was of 261. Importantly, there is no crisis discharge in this unit since psychiatric admissions cannot be refuted and number of beds is usually sufficient to cover the needs of acute care. In the rare cases of bed lacking, the hospital stays take place in an adult psychiatry unit. Patients are admitted to the UHPP in the presence of acute symptoms associated with self or others-threatening behavior and need for urgent psychiatric care. In order to focus on long-term determinants, we excluded all of the cases with hospitalisations due to treatment discontinuation or active drug addiction. All of the ICD-10 clinical diagnoses were made prospectively by two independent, board-certified psychiatrists (prior and during the hospital stay), blind to the scope of the study. The inter-rater reliability was high (kappa value of 0.88). Only cases with concordant psychiatric diagnoses were considered in this sample. After applying these two criteria, the intermediate sample was of 620 cases. We randomly selected 200 cases to form the final sample of the study (mean age: 32.8 ± 10.3, age range: 20–44). Three levels of admission were considered: single, frequent, RD. Their definition was made according to the number of admissions during a 12-months interval within the period of reference. According to this definition, there were 100 single, 69 frequent and 31 RD users that were considered for further statistical analysis. Among single users, 62% were admitted voluntarily. This percentage was of 32% in frequent users (with 40% of mixed and 28% of compulsory only admissions). In RD users, the corresponding percentages were of 28, 41, and 31% respectively. The study was performed in agreement with the declaration of Helsinki and approved by the ethical Committee of Geneva, Switzerland. In order to guarantee confidentiality, all data were treated anonymously.

Sociodemographic data, ICD-10 clinical diagnosis, psychiatric history including outpatient care and previous inpatient stays prior to incarceration were recorded. Drug misuse was considered as binary variable and coded as present/absent. Suicidality (presence of suicidal behaviors and suicidal thoughts) was also treated as binary variable. The criminal status was coded as ordinary detention (remand or after sentence), detention with compelling psychiatric outpatient treatment (art 63 Swiss Criminal Code) or compelling institutional treatment (art 59, 60 Swiss Criminal Code).

### Statistical Analysis

The Cuzick's test for trend across ordered groups (29), Kruskal-Wallis test and oneway ANOVA were used to compare demographic and clinical variables between admissions' type (single, frequent and RD). In addition, univariate and multiple ordered logistic regression models were built to examine the determinants of the 3 levels of admissions' type. All statistical analysis were performed using Stata 16.1.

## Results

There were no age and gender differences between single, frequent and RD users. The three groups did not differ in respect to the proportion of cases with fluent French speaking, education and marital status (Table 1).

**Table 1:** Characteristics of inpatients by type of admission.

	**Single**	**Frequent**	**RD**	**Total**	***P***
N	100	69	31	200	
Age	33.0 ± 10.7	33.6 ± 11.0	30.0 ± 6.5	32.8 ± 10.3	0.246
Sex women	14 (14.0%)	9 (13.0%)	4 (12.9%)	27 (13.5%)	0.840
Language French	76 (76.0%)	51 (73.9%)	28 (90.3%)	155 (77.5%)	0.296
Education more than 9 years	21 (21.0%)	15 (21.7%)	7 (22.6%)	43 (21.5%)	0.847
**Marital status**					0.260
Married	17 (17.0%)	16 (23.2%)	3 (9.7%)	36 (18.0%)	
Separed-divorced-widowed	19 (19.0%)	13 (18.8%)	3 (9.7%)	35 (17.5%)	
Single	64 (64.0%)	40 (58.0%)	25 (80.6%)	129 (64.5%)	
**Criminal status**					<0.001
Usual detention	84 (84.0%)	47 (68.1%)	8 (25.8%)	139 (69.5%)	
Court ordered treatments	16 (16.0%)	22 (31.9%)	23 (74.2%)	61 (30.5%)	
Psychiatric outpatient care	73 (73.0%)	54 (78.3%)	29 (93.5%)	156 (78.0%)	0.032
Psychiatric inpatient care	55 (55.0%)	31 (47.7%)	23 (76.7%)	109 (55.9%)	0.298
Suicidal behavior	39 (39.0%)	33 (47.8%)	19 (61.3%)	91 (45.5%)	0.033
Adjustment disorder	29 (29.0%)	10 (14.5%)	0 (0.0%)	39 (19.5%)	<0.001
Bipolar disorder	4 (4.0%)	4 (5.8%)	1 (3.2%)	9 (4.5%)	0.884
Depressive disorder	10 (10.0%)	4 (5.8%)	0 (0.0%)	14 (7.0%)	0.057
Personality disorder	22 (22.0%)	23 (33.3%)	14 (45.2%)	59 (29.5%)	0.010
Psychotic disorder	32 (32.0%)	28 (40.6%)	16 (51.6%)	76 (38.0%)	0.047

Single users were admitted voluntarily in 64% of cases, this percentage being 31% in frequent and only 4.4% in RD users (*p* < 0.001). With respect to criminal status, 139 patients (69.5%) were detained under usual detention conditions (pre-trial detention or conviction after judgement), whereas 61 patients (30.5%) were under court-ordered treatment. RD was significantly more frequent in patients with court-ordered treatment (*p* < 0.001). In fact, 74.2% of RD cases had this latter criminal status compared to only 16% of single users. Previous outpatient psychiatric care was also significantly more frequent among RD users compared to the two other groups (*p* = 0.032). The percentage of cases with previous hospital stay reached 76.7% of RD users, compared to 55% of single and 47.7% of frequent users. This difference did not reach statistical significance. RD users showed significantly higher rates of suicidal behavior (61.3%) compared to single (39%) users (*p* = 0.033). The ICD-10 diagnoses of personality and psychotic disorders were significantly more frequent in RD cases compared to the other groups (*p* = 0.01 and 0.047, respectively). In contrast, no RD case had a diagnosis of adjustment disorders (*p* < 0.001). No group difference was found in the percentage of bipolar and depressive disorders (Table 1).

In univariate ordered logistic regression models taking into account the socio-demographic variables, criminal status and clinical diagnosis, RD status was positively associated with court ordered treatments, psychiatric outpatient care, personality and psychotic disorders with OR ranging from 1.737 to 5.774. A negative relationship was found between RD and the diagnosis of adjustment disorders (OR = 0.246). In multivariable models, only the associations with court-ordered treatments [OR = 5.93, 95% CI (2.76, 12.75), *p* < 0.001] and personality disorder [OR = 2.87 (1.03, 7.98), *p* = 0.044] persisted (Table 2).

**Table 2:** Results of univariate (unadjusted OR) and multiple (adjusted OR) ordered logistic regression associated with more frequent admissions (3 levels).

	**Unadjusted**		**Adjusted**	
**Characteristics**	**Odds ratio**	***P***	**Odds ratio**	***P***
Age	0.989 (0.965, 1.015)	0.408	0.979 (0.945, 1.015)	0.250
Sex women	1.084 (0.499, 2.354)	0.839	0.827 (0.346, 1.974)	0.668
Language French	1.389 (0.741, 2.604)	0.306	1.436 (0.693, 2.975)	0.330
Education more than 9 years	1.065 (0.562, 2.020)	0.847	0.696 (0.326, 1.487)	0.349
**Marital status**				
Married	0.882 (0.444, 1.751)	0.719	1.935 (0.875, 4.278)	0.103
Separed-divorced-widowed	0.715 (0.350, 1.460)	0.357	0.811 (0.326, 2.015)	0.652
Single	1.000 (1.000, 1.000)		1.000 (1.000, 1.000)	
**Criminal status**				
Usual detention	1.000 (1.000, 1.000)		1.000 (1.000, 1.000)	
Court ordered treatments	5.774 (3.100, 10.757)	<0.001	6.032 (2.816, 12.922)	<0.001
Psychiatric outpatient care	2.028 (1.049, 3.920)	0.035	1.667 (0.683, 4.067)	0.262
Psychiatric inpatient care	1.330 (0.776, 2.281)	0.299	0.622 (0.294, 1.318)	0.215
Adjustement disorder	0.246 (0.114, 0.531)	<0.001	0.602 (0.183, 1.982)	0.404
Personality disorder	2.139 (1.200, 3.811)	0.010	2.867 (1.030, 7.980)	0.044
Psychotic disorder	1.737 (1.008, 2.992)	0.047	1.975 (0.716, 5.445)	0.188

## Discussion

### Summary

Our data show that RD use is associated with the criminal status, psychiatric history of outpatient care prior to the conviction, as well as presence of personality and psychotic disorders. When demographic, social and clinical parameters were taken into account simultaneously in multivariable regression models, criminal status and presence of personality disorders were the only independent determinants of RD in prison inmates.

In the absence of previous studies regarding RD use in psychiatric settings for forensic patients, one should compare these observations with those made in adult psychiatry. From a methodological viewpoint, there are some main differences between our study and those referring to non-incarcerated patients. First, drug addiction was very rare due to the stringent control of drug consumption in the Swiss penitentiary system. Second, cases with drug discontinuation causing hospital stays were excluded from the present analysis in order to focus on long-term determinants of RD in forensic patients. Third, policy-related causes of RD had minimal impact in this particular setting. In fact, the number of acute psychiatric beds for inmates, that is known to be a main determinant of RD in adult psychiatry settings for non-inmates, was sufficiently high to prevent for crisis discharges (10, 11). Fourth, the efficacy of community care system, known to be another determinant of (3, 7, 30, 31) does not concern this population. Last but not least, the small percentage of women (only 13.5%) does not allow for drawing definite conclusions about a possible gender-related vulnerability to RD in inmates (32).

### Revolving Door and Demographic Factors

No demographic factor was associated with RD in the present study. Young age has been previously associated with RD in non-inmates possibly because of increased impulsiveness and low adherence to outpatient care (7, 22, 31, 33, 34). Such age difference was not observed in the present sample but one should take into account its narrow age distribution. Although the age range was between 20 and 43 years, 70% of patients were younger than 35 years. In the same line, there was no significant difference in RD frequency as a function of the level of education. Heavy users of psychiatric services in community-based settings are usually poorly educated [maximum of middle school degree; (34)]. In our series, less that 22% of patients had a high school level in all three groups pointing to a floor effect due to the social disadvantage usually observed in penitentiary populations. Rabinowitz et al. (35) found that being married at the time of the first hospitalization is a strong protective factor for RD. Similar observations were made later on (30) pointing to the vulnerability of single men to RD. Most of the RD patients were single (80%), yet the difference observed compared to single and frequent users (64 and 58%, respectively), remained non-significant. Previous inpatient care is thought to be one among the strongest determinants of readmission in non-inmates (12, 31). Data are still missing for outpatient care in this population, yet the frequent occurrence of RD among patients with chronic and severely debilitating mental illness followed-up in the community (17–21) implies that this parameter is, at least, indirectly associated with RD. In our sample, the frequency of previous inpatient care was clearly higher in RD users (76.7%) compared to the two other groups (55 and 47.7%, respectively), yet this difference did not reach statistical significance. In contrast, outpatient psychiatric care prior to incarceration is associated with increased RD use in prison. This observation points to a prison-independent mental health burden already present in RD users.

As one can expect and in line with early observations in non-inmates, suicidal behavior was more frequent in RD cases (15, 16). However, this parameter is obviously not a stable long-term determinant of RD use. In contrast, the presence of court-ordered treatments in place of usual conviction was positively associated with RD both in univariate and multivariable models with an OR close to 6. This finding suggests that this criminal status reflects a long-lasting psychological vulnerability that renders acute psychiatric care more frequently needed compared to other inmates.

### Revolving Door and Clinical Diagnosis

The most challenging observations concern the association between clinical diagnosis and RD use. Using both group comparisons and univariate regression models, we document the positive association between personality as well as psychotic disorders and RD use. This observation parallels the data reported in non-inmates in respect to schizophrenia and heavy use of psychiatric inpatient wards (7, 36, 37). This disorder is related to severe outcome, poor insight and low treatment adherence that are thought to favor RD (7, 13). However, the association between schizophrenia and RD in non-inmates was mostly present in patients with comorbid drug addiction (13). Our data show that in prison, this association persists even in the absence of active drug addiction and treatment discontinuation. Previous studies also reported a positive association between RD use and bipolar illness (14) not found in our sample. Yet, very limited number of bipolar cases in all three groups prevents from drawing definite conclusions on this matter. Patients with borderline personality disorders were frequently described as heavy users of psychiatric inpatient care in the community (33, 34) whereas no data are available for the other personality disorders. More recently, Di Lorenzo et al. (5) also pointed to a global association between personality and RD in community-based samples. In the present series, we found a strong relationship between the presence of personality disorders and RD that persisted in multivariate models with an OR of 2.87. Given the limited number of cases with such diagnosis and for keeping statistical power, no separate analysis was made according to the type of personality disorder. It is however noteworthy that the negative impact of personality disorders persisted after adjusting for all of the other variables indicating an independent role of personality in the prediction of RD in prison inmates. Last but not least, the diagnosis of adjustment disorder was absent in RD inmates whereas it was made in almost 30% of single users. Taken together, these findings suggest that acute psychiatric care in these patients did not depend of environmental stressors but rather represents the expression of a long-lasting vulnerability related to their psychological profile and criminal status.

### Strengths and Limitations

Strengths of the present study includes the presence of a single unit of hospital stay that decreases the variability in the criteria of admission, exclusion of cases with severe drug addiction and drug discontinuation, stringent definition of RD that correspond to a massive utilization of psychiatric inpatient care, and use of multivariable models that make it possible to control for the interdependence between the clinical, forensic and socio-demographic variables. Some limitations should, however, be considered. First, to be close to a real life situation, clinical diagnosis was made by two independent clinicians blind to the scope of the study but without use of standardized diagnostic questionnaires. Second, the definition of frequent and RD users was based on the number of admissions during a 12-month period and did not take into account the length of the hospital stay. We cannot thus comment on the determinants of the heavy use of psychiatric inpatient care in prison. Third, given the limited number of cases and presence of mixed patterns of admission (voluntary and compulsory) among frequent and RD users, we cannot draw conclusions regarding the association between admission type and RD in our sample. Lastly, our results cannot be generalized since the prison system in Switzerland differs from that of other European countries in terms of bed availability for inpatient psychiatric care of inmates. Future studies in larger inmate samples from different countries including standardized assessment of the clinical diagnosis taking into account the cumulative length of hospital stays are needed to explore the determinants and economic consequences of RD in inmates with psychiatric disorders.

## Data Availability Statement

The raw data supporting the conclusions of this article will be made available by the authors, without undue reservation.

## Ethics Statement

Ethical approval for this study was obtained by the local Ethic Committee. Written informed consent for participation was not required for this study in accordance with the national legislation and the institutional requirements.

## Author Contributions

ID'O and PG contributed to the conception, design of the study, and wrote the paper. ID'O was involved in the acquisition of data and supervision of the database. FH performed the statistical analysis. All authors contributed to manuscript revision and approved the submitted version.

## Conflict of Interest

The authors declare that the research was conducted in the absence of any commercial or financial relationships that could be construed as a potential conflict of interest.
